# Examining the Polymorphisms in the Hypoxia Pathway Genes in Relation to Outcome in Colorectal Cancer

**DOI:** 10.1371/journal.pone.0113513

**Published:** 2014-11-18

**Authors:** Asan M. S. Haja Mohideen, Angela Hyde, Jessica Squires, Jing Wang, Elizabeth Dicks, Ban Younghusband, Patrick Parfrey, Roger Green, Sevtap Savas

**Affiliations:** 1 Discipline of Genetics, Faculty of Medicine, Memorial University of Newfoundland, St. John's, NL, Canada; 2 Division of Community Health and Humanities, Faculty of Medicine, Memorial University of Newfoundland, St. John's, NL, Canada; 3 Clinical Epidemiology Unit, Faculty of Medicine, Memorial University of Newfoundland, St. John's, NL, Canada; 4 Discipline of Oncology, Faculty of Medicine, Memorial University of Newfoundland, St. John's, NL, Canada; Medical University of Graz, Austria

## Abstract

**Introduction:**

Colorectal cancer is a common malignancy. Identification of genetic prognostic markers may help prognostic estimations in colorectal cancer. Genes that regulate response to hypoxia and other genes that are regulated under the hypoxic conditions have been shown to play roles in cancer progression. In this study, we hypothesized that genetic variations in the hypoxia pathway genes were associated with the risk of outcome in colorectal cancer patients.

**Methods:**

This study was performed in two phases. In the first phase, 49 SNPs from six hypoxia pathway genes (*HIF1A*, *HIF1B*, *HIF2A*, *LOX*, *MIF* and *CXCL12*) in 272 colorectal cancer patients were analyzed. In the second phase, 77 SNPs from seven hypoxia pathway genes (*HIF1A*, *HIF1B*, *HIF2A*, *HIF2B*, *HIF3A*, *LOX* and *CXCL12*) were analyzed in an additional cohort of 535 patients. Kaplan Meier, Cox univariate and multivariable regression analyses were performed to analyze the relationship between the SNPs and overall survival (OS), disease free survival (DFS) or disease specific survival (DSS). Since this was a hypothesis-generating study, no correction for multiple testing was applied.

**Results:**

In phase I, one SNP (*HIF2A* rs11125070) was found to be associated with DFS in multivariable analysis; yet association of a proxy polymorphism (*HIF2A* rs4953342) was not detected in the phase II patient cohort. In phase II, associations of two SNPs (*HIF2A* rs4953352 and *HIF2B* rs12593988) were significant in both OS and DFS multivariable analyses. However, association of *HIF2A* rs4953352 was not replicated in the phase I cohort using a proxy SNP (*HIF2A* rs6706003).

**Conclusion:**

Overall, our study did not find a convincing evidence of association of the investigated polymorphisms with the disease outcomes in colorectal cancer.

## Introduction

Hypoxia is a condition characterised by low oxygen levels. Solid tumour cells may experience hypoxic conditions due to restricted blood flow. While this may cause reduced cell proliferation or death, sometime it also helps cells adapt to hypoxic conditions by altering their energy metabolism from oxidative phosphorylation pathway to glycolysis pathway. Such alterations influence the expression of hypoxia-inducible genes and treatment outcome in cancer patients. In addition, hypoxic conditions have been implicated to promote DNA replication, angiogenesis, and tumor invasion and metastatic potential. All of these changes facilitate tumor progression and may negatively affect the patient outcome. These and other roles of hypoxic conditions in tumor progression and outcome have been extensively reviewed by many in literature (for example, [1], [2]).

Under hypoxic conditions, cells activate specific molecular machineries by up-regulating or down-regulating the expression of certain genes. This is facilitated by the hypoxia inducible factors (HIFs). HIFs are heterodimeric transcription factors consisting of α and β subunits. In humans, there are three HIF-α (HIF-1α, HIF-2α and HIF-3α) and two HIF-β (HIF-1β and HIF-2β). Each of these subunits is coded by distinct genes (*HIF1A*, *ARNT*/*HIF1B*, *EPAS1*/*HIF2A*, *ARNT2*/*HIF2B*, and *HIF3A*). HIFs bind to hypoxia responsive elements (HREs) along the hypoxia-regulated genes to regulate their expression. These hypoxia-inducible genes include genes functioning in cell growth, metabolism, DNA damage response, angiogenesis, and metastasis (reviewed in [2], [3]). Moreover, HIFs also activate genes functioning in cellular mechanisms that lead resistance to conventional anti-cancer therapies (reviewed in [3]).

Among the genes regulated by the HIFs are the lysyl oxidase (*LOX*) [4], macrophage migration inhibitory factor (*MIF*) [5] and C-X-C motif chemokine 12 (*CXCL12*) [6]. *LOX* codes for an enzyme that helps maintain the structural integrity of the connective tissue and has been identified as a critical driver of the hypoxia-induced metastasis in human breast tumors [7]. MIF is known predominantly as an immune system protein, yet in colon cancer cell lines it promotes hypoxia-driven apoptosis [8]. CXCL12 is another protein mostly known for its role in the immune system, however it has been shown to influence the tumor cell death and reduce the metastasis risk in colorectal tumor cell lines [9].

Colorectal cancer is a common cancer in developed countries. In Canada, according to the Canadian Cancer Society Statistics-2012, it is one of the leading causes of cancer related mortalities [10]. Currently established markers are insufficient for accurate prediction of prognosis in colorectal cancer patients. Therefore, identification of new prognostic markers may assist improving the prognostic models, which in turn may help improve the survival outcomes of colorectal cancer patients. In this study, we hypothesized that the genetic variations within the select genes of the hypoxia pathway are associated with the risk of outcome in colorectal cancer patients. To test our hypothesis, we conducted this study in two phases: In phase I, we focused on three HIF-coding genes (*HIF1A*, *HIF1B*, and *HIF2A*) and three genes regulated under the hypoxic conditions (*LOX*, *MIF*, and *CXCL12*) and investigated the relationship of their SNPs (n = 49) with outcomes in a small cohort of colorectal cancer patients (n = 272). In phase II, we focused on five HIF-coding genes (*HIF1A*, *HIF1B*, *HIF2A*, *HIF2B* and *HIF3A*) and two hypoxia-inducible genes (*LOX* and *CXCL12*) and investigated the relationship of their SNPs (n = 77) with the risk of outcome in an additional colorectal cancer patient cohort (n = 535).

## Materials and Methods

**Figure 1:**
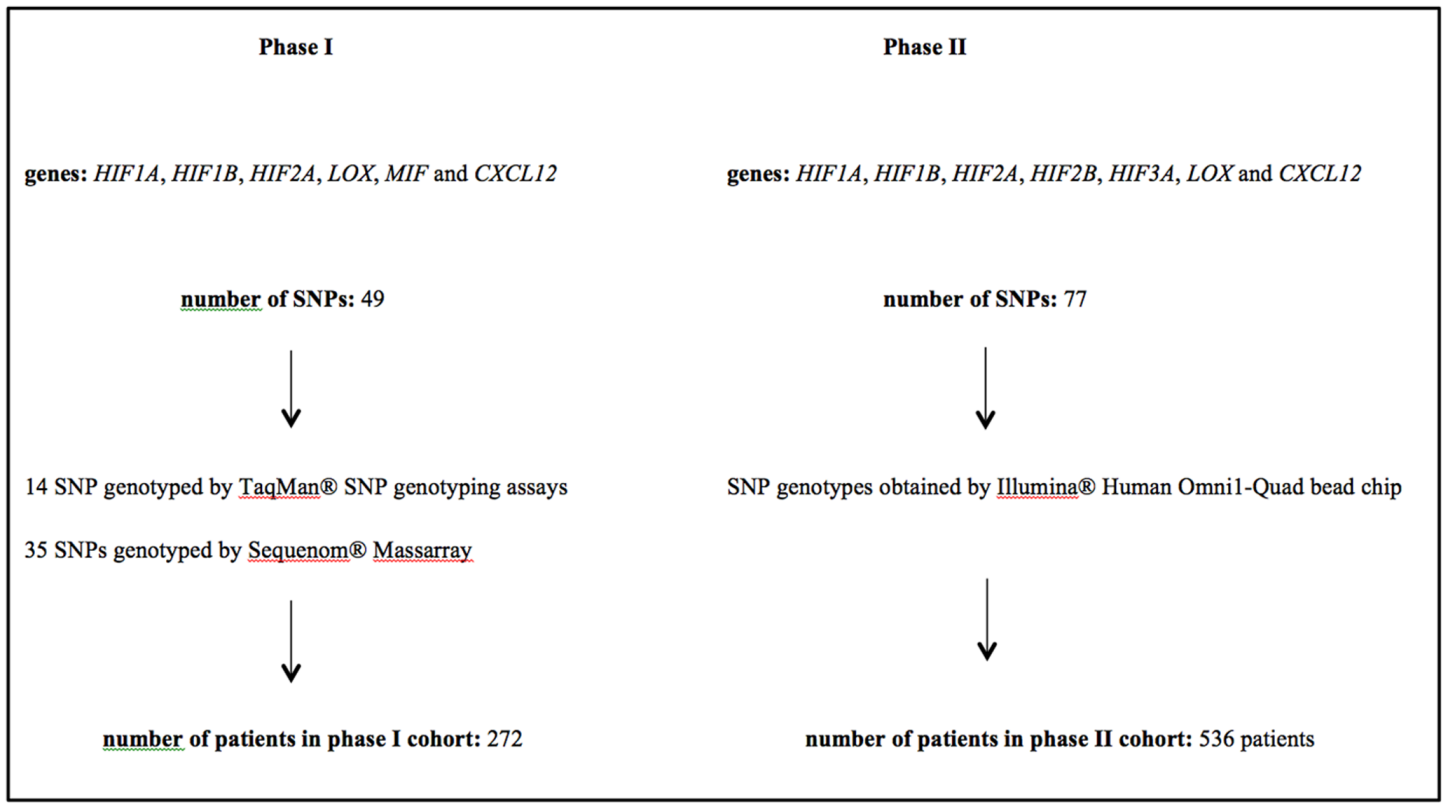
SNP: single nucleotide polymorphism.

This research project was carried out in two phases: phase I and phase II. Phase II was initiated after the completion of phase I when a large-scale genotype data for a larger patient cohort was obtained by our group as part of another project. As illustrated in **Figure 1**, there are differences between phase I and phase II in terms of genes, SNPs and patient cohorts investigated.

### Ethic statement

Requirement for patient consent was waived by the local REB committee (Human Investigation Committee (HIC) of Memorial University; recently renamed as The Health Research Ethics Authority (HREA)) for the patients in phase I. Written consent was obtained from either the patients or their family members (in case of deceased patients) in the phase II cohort. During this study, all patient-related data was investigated anonymously. This particular study was also approved by HIC.

### Study samples

#### a) Phase I cohort

The first cohort consisted of 280 patients and was described in detail previously [11]. These patients were diagnosed with colorectal cancer between 1997–1998 in the Avalon Peninsula, Newfoundland. Patient in this cohort were followed up till 2009. For this project, DNA samples from 272 of the patients were available for the genotyping reactions.

#### b) Phase II cohort

The second cohort is a sub-cohort of the patients recruited to the Newfoundland Colorectal Cancer Registry (NFCCR). The NFCCR cohort was recruited between 1999 and 2003 and described in other publications [12], [13]. In the NFCCR cohort, there are 736 patients with stage I–IV tumors and with clinicopathological and prognostic data collected till 2010 [11]. Among these patients, a total of 535 patients with available genotypes obtained using the genomewide SNP genotyping method (*see below*) were included in this phase of the project.

### Selection of genes

#### a) Phase I

Six hypoxia pathway genes (*HIF1A*, *HIF1B*, *HIF2A*, *LOX*, *MIF* and *CXCL12*) were selected.

#### b) Phase II

In phase II of this project, our primary aim was to investigate the associations of polymorphisms from the selected genes in phase I (*HIF1A*, *HIF1B*, *HIF2A*, *LOX*, *MIF* and *CXCL12*) in a larger patient cohort. By taking advantage of the availability of genotypes, we also aimed to include two additional HIF-coding genes (*HIF2B* and *HIF3A*) in this phase.

### Selection of SNPs

#### a) Phase I

In order to prevent redundancy in polymorphisms investigated, we followed an approach that involved the calculation of correlation coefficients (r^2^) between the genotypes of polymorphisms per gene; from those SNPs that were highly correlated with each (r^2^≥0.8), only one representative SNP was included into the study.

For this purpose, for each gene included in this study the genotype data for the Caucasian samples were downloaded from the HapMap database [14] prior to start of the project, which were used to construct linkage disequilibrium maps of the genes using the Haploview software [15]. r^2^ values were calculated and tagSNPs were determined using the pairwise tagger [16] procedure implemented in Haploview. Both tagSNPs and SNPs that are not tagged by the tagSNPs were aimed to be included to have a comprehensive analysis of each gene. In phase I, total of 49 such SNPs were successfully genotyped using this approach (**Table S1 in File S1**). Among the 49 SNPs, *HIF2A* rs2346175 polymorphism had >15% missing data and three polymorphisms (*HIF1B* rs3738483, *HIF2A* rs6753127 and *HIF2A* rs11687512) had minor allele frequencies (MAFs)<10% in the phase I cohort.

#### b) Phase II

Eighty-one SNPs were selected from the eight hypoxia pathway genes using the approach described in phase I. From the selected SNPs, four SNPs that had a MAF<10% were excluded from the statistical analysis (*HIF1B* rs10305724, *HIF1B* rs3738483, *HIF2B* rs16972160, and *HIF2B* rs1139651), which resulted in 77 SNPs to be included in this phase (**Table S2 in File S1**). No polymorphism had more than 15% missing genotype data. Genotypes of no SNP from the *MIF* gene was available for this cohort. Thus, a total of 77 SNPs from seven genes (*HIF1A*, *HIF1B*, *HIF2A*, *HIF2B*, *HIF3A*, *LOX* and *CXCL12*) were included into phase II.

Thirteen SNPs were investigated in both patient cohorts. In addition, there were 15 SNPs investigated in phase I that had highly correlated genotypes with other SNPs investigated in the phase II cohort (**Table S3 in File S1**): the remaining SNPs were investigated in either cohort I or cohort II, but not in both. Since SNPs with highly correlated genotypes can serve as surrogates for each other, during this study we also checked (in addition to identical SNPs) whether the results of the statistical tests obtained for proxy SNPs in both cohorts were similar in terms of their associations with the survival times.

### Genotyping

#### a) Phase I

In this phase, DNA samples were extracted either from blood samples or from non-tumor colorectal tissue blocks obtained during surgery. The genotypes of the 49 polymorphisms included in this phase of the study were obtained by either Sequenom MassArray technology at an outsourcing genotyping facility (University Health Network Analytical Genetics Technology Centre, Canada; n = 35 SNPs) or in-house TaqMan SNP genotyping assays (n = 14 SNPs). For both MassArray and TaqMan SNP genotyping assays, at least 5% of the study subjects were genotyped twice and all genotypes obtained were 100% concordant. Each genotype reaction also contained non-template controls to detect external DNA contamination. Those DNA samples that were failed to be genotyped by TaqMan SNP genotyping assays were attempted to be genotyped two or more times depending on the availability of DNA samples.

### TaqMan SNP genotyping

TaqMan SNP genotyping assays were performed in a 96 well fast reaction plates using the ABI 7900HT Fast Real-Time PCR System. Typically genotyping reactions contained 9 µl of reaction mix and 1 µl of DNA sample (4 ng/µl). The reaction mix consisted of 5 µl of TaqMan Universal PCR Master Mix (2×) (Applied Biosystems PN 4304437), 0.25 µl of SNP Genotyping Assay Mix (20×) (specific to each SNP) and 3.25 µl of sterile water. In some cases, especially those that showed poor amplification, the reaction volume was 5 µl (containing 4 ng DNA); this was done to increase the DNA concentrations in reactions. The assay IDs for the SNPs genotyped by this method are shown in **Table S4 in File S1**. A pre-run scan was performed prior to start of amplification. The PCR reaction conditions were as follow: a) activation of AmpErase UNG at 50°C for 2 mins, b) AmpliTaq Gold polymerase activation at 95°C for 10 mins, and c) 40 cycles of denaturation of DNA at 95°C for 15 sec followed by primer annealing and extension at 60°C for 1 min. After completion of the reactions, a post-run scan was performed and the data was analyzed using the sequence detection software (SDS). The SDS genotyping results were also manually checked to call the final genotypes by one of us (SS).

#### b) Phase II

In phase II of this project, the genotype data of the 77 SNPs were obtained as a part of a whole genome SNP genotyping study. Genotypes were obtained using the Illumina Human Omni1-Quad Bead Chip at a service provider (Centrillion Genomic Services, USA) using the DNA samples extracted from the blood samples.

### Statistical methods

Genotypes obtained were organised in Microsoft Excel sheets and the statistical analyses were performed using the Statistical Package for Social Sciences (SPSS) software. Prior to statistical analysis, all variables were checked for missing data. In addition, MAFs of SNPs were calculated and genotypes were checked for deviations from the Hardy Weinberg Equilibrium (HWE). HWE was calculated using the Chi-square test. Variables that had more than 15% missing data or deviating from the HWE were included into the univariate analysis for exploratory purposes, but were excluded from the multivariable analysis. Genotypes were coded assuming the dominant genetic model. Except age, all other variables included in the analysis were categorical; age was analysed as a continuous variable.

Three different measures of outcome were used for the statistical analysis: overall survival (OS), disease free survival (DFS) and disease specific survival (DSS). For OS, death was the clinical end point (defined as death by any cause). For DFS, occurrence of recurrence of disease or metastasis or death was the clinical end point. For DSS, colorectal cancer specific death was the clinical end point. DSS information was available only for phase I cohort. Patients who did not experience the event of interest during the follow up period were censored at the date of their last follow up.

Survival curves were generated by the Kaplan Meier method. The relationship between each variable and the outcome measures (OS, DFS, DSS) was analyzed individually using Cox regression method in univariate analysis. The p-values, Hazard Ratios (HRs) and the 95% Confidence Intervals (CIs) for the HRs were also computed by the Cox regression method. Variables that were statistically significant in the univariate analysis (p<0.05) were included into the multivariable Cox regression models. The patient characteristics between two study cohorts were compared using the Chi-square test statistic for categorical variables and the Mann-Whitney U test for the continuous variables. A p value less than 0.05 was considered as statistically significant; as this was an exploratory analysis no correction for multiple testing was performed. All tests were double sided.

## Results

### Phase I

Baseline characteristics of the phase I cohort are shown in **Table 1**. In this cohort (n = 280), the median age at diagnosis was 68.4 years (range: 25.3–91.6), the median OS and DSS follow up time was 5.3 years (range: 0–12.5 years) and the median DFS follow up time was 3.4 years (range: 0–12.5 years).

**Table 1:** Baseline characteristics of the phase I cohort.

Variables	n	%
**Sex**		
Male	150	53.6
Female	130	46.4
**Age at diagnosis**		
Median	68.42 years (range:25.29–91.61)	
**Grade**		
Poorly differentiated/undifferentiated	42	15
Well/moderately differentiated	234	83.6
Unknown	4	1.4
**Histology**		
Mucinous	43	15.4
Non-mucinous	237	84.6
**Location**		
Rectum	57	20.4
Colon	223	79.6
**Lymphatic invasion of tumor**		
Lymphatic invasion (+)	110	39.3
Lymphatic invasion (−)	70	25
Unknown	100	35.7
**Stage**		
I	54	19.3
II	94	33.6
III	76	27.1
IV	47	16.8
Unknown	9	3.2
**MSI status**		
MSI-H	34	12.1
MSS/MSI-L	246	87.9
**Prognostic and follow up information**		
**OS status at the time of last follow up**		
Dead	172	61.4
Alive	108	38.6
Median OS and DSS (follow up) time	5.31 years (range: 0–12.52)	
**DFS status at the time of last follow up**		
Recurrence/metastasis/death (+)	184	65.7
Recurrence/metastasis/death (−)	96	34.3
DFS (follow up) time	3.37 years (range: 0–12.52)	
**DSS status at the time of last follow up**		
Death from colorectal cancer	113	40.4
Death from other causes or alive	167	59.6

(+): present, (−): absent, DFS: disease free survival, DSS: disease specific survival, MSI-H: microsatellite instability-high, MSI-L: microsatellite instability-low, MSS: microsatellite stable, OS: overall survival.

Out of 49 polymorphisms investigated in this phase, the genotype frequencies for seven SNPs (*LOX* rs10040971, *HIF1B* rs10847, *CXCL12* rs2236534, *CXCL12* rs2236533, *CXCL12* rs11592974, *HIF2A* rs9973653 and *HIF2A* rs4145836) deviated from HWE (**Table S1 in File S1**). These SNPs were included in univariate analysis for exploratory purposes.

In univariate analysis for overall survival, three SNPs were found to be significantly associated (p<0.05) with outcome: *LOX* rs10519694 (p = 0.046; HR = 0.735; 95% CI: 0.543–0.994), *HIF2A* rs11125070 (p = 0.003; HR = 0.616; 95% CI: 0.447–0.848) and *HIF2A* rs1868084 (p = 0.024; HR = 0.678; 95% CI: 0.483–0.950; **Table S5 in File S1**). However, in a multivariable model, associations of none of these SNPs remained statistically significant when adjusted for age, grade, stage and MSI status (**Table 2**).

**Table 2:** Multivariable analysis results for overall survival (phase I; n = 234).

Variables	p-value	HR	95% CI (lower)	95% CI (upper)
*LOX* rs10519694 (CT+TT vs CC)	0.129	0.767	0.544	1.08
*HIF2A* rs11125070 (AT+TT vs AA)	0.282	0.801	0.535	1.2
*HIF2A* rs1868084 (GC+GG vs CC)	0.293	0.796	0.521	1.218
Age	**<0.001**	1.045	1.029	1.06
Grade (poorly differentiated/undifferentiated vs well/moderately differentiated)	**<0.001**	2.594	1.669	4.03
Stage	**<0.001**			
Stage (II vs I)	0.247	1.399	0.792	2.472
Stage (III vs I)	**0.002**	2.534	1.423	4.514
Stage (IV vs I)	**<0.001**	14.398	7.617	27.213
MSI status (MSI-H vs MSS/MSI-L)	**0.002**	0.285	0.131	0.62

CI: confidence interval, HR: hazard ratio, MSI-H: microsatellite instability-high, MSI-L: microsatellite instability-low, MSS: microsatellite stable. Significant associations (p<0.05) are shown in bold.

In univariate analysis for disease specific survival, association of none of the SNPs were significant (**Table S6 in File S1**).

In univariate analysis for disease free survival, two SNPs (*LOX* rs10519694; p = 0.012; HR = 0.685; 95% CI: 0.510–0.919 and *HIF2A* rs11125070; p = 0.003; HR = 0.629; 95% CI: 0.461–0.858) were associated (p<0.05) with the survival time (**Table S7 in File S1**). One of these SNPs (*HIF2A* rs11125070) remained statistically significant in the multivariable analysis when adjusted for *LOX* rs10519694 genotypes, age, grade, stage, and MSI status (HR: 0.619, 95% CI: 0.446–0.859, p = 0.004; **Table 3**).

**Table 3:** Multivariable analysis results for disease free survival (phase I; n = 236).

Variables	p-value	HR	95% CI (lower)	95% CI (upper)
*LOX* rs10519694 (CT+TT vs CC)	0.113	0.767	0.552	1.065
*HIF2A* rs11125070 (AT+TT vs AA)	**0.004**	0.619	0.446	0.859
Age	**<0.001**	1.034	1.019	1.048
Grade (poorly differentiated/undifferentiated vs well/moderately differentiated)	0.099	1.437	0.934	2.213
Stage	**<0.001**			
Stage (II vs I)	0.112	1.553	0.903	2.673
Stage (III vs I)	**<0.001**	2.93	1.684	5.095
Stage (IV vs I)	**<0.001**	133.705	55.984	319.324
MSI status (MSI-H vs MSS/MSI-L)	**0.013**	0.4	0.194	0.825

CI: confidence interval, HR: hazard ratio, MSI-H: microsatellite instability-high, MSI-L: microsatellite instability-low, MSS: microsatellite stable, n: number of patients. Significant associations (p<0.05) are shown in bold.

### Phase II

After the completion of phase I, a more comprehensive study (phase II) was performed by adding polymorphisms from two more HIF-coding genes (*HIF2B*, *HIF3A*) and by investigating their associations with OS and DFS in a second and larger patient cohort.

Baseline characteristics of the phase II cohort are shown in **Table 4**. In the phase II cohort (n = 535), the median age was 61.2 years (range: 20.7–75.0 years), the median OS time was 6.34 years (range: 0.38–10.88) and the median DFS time was 5.98 years (range: 0.22–10.88).

**Table 4:** Baseline characteristics of the phase II cohort.

Variables	n	%
**Sex**		
Female	207	38.7
Male	328	61.3
**Age at diagnosis**		
Median	61.23 years (range: 20.7–75)	
**Histology**		
Mucinous	61	11.4
Non-mucinous	474	88.6
**Location**		
Colon	355	66.4
Rectum	180	33.6
**Stage**		
I	97	18.1
II	207	38.7
III	178	33.3
IV	53	9.9
**Grade**		
Well/moderately differentiated	492	92
Poorly differentiated/undifferentiated	39	7.3
Unknown	4	0.7
**Vascular Invasion**		
Vascular Invasion (−)	325	60.7
Vascular Invasion (+)	171	32
Unknown	39	7.3
**MSI status**		
MSI-H	58	10.84
MSS/MSI-L	455	85.05
Unknown	22	4.11
**Prognostic and follow up information**		
**OS status**		
Alive	352	65.8
Dead	182	34.02
Unknown	1	0.18
**OS time (follow up time)**		
Median	6.34 years (range 0.38–10.88)	
**DFS status**		
Recurrence/metastasis/death (−)	321	60
Recurrence/metastasis/death (+)	213	39.8
Unknown	1	0.2
**DFS time (follow up time)**		
Median	5.98 years (range: 0.22–10.88)	

(+): present, (−): absent, DFS: disease free survival, MSI-H: microsatellite instability-high, MSI-L: microsatellite instability-low, MSS: microsatellite stable, OS: overall survival.

Of 77 SNPs (**Table S2 in File S1**), genotype frequencies of seven polymorphisms deviated from HWE (*HIF2B* rs8041826, *HIF2B* rs7172914, *HIF2B* rs1020398, *HIF2B* rs4778600, *HIF2B* rs8033706, *HIF3A* rs12461322 and *HIF3A* rs11665853); these SNPs were included only in the univariate analysis for exploratory purposes.

In this phase of the project, *HIF2A* rs4953352 (p = 0.012; HR = 1.596; 95% CI: 1.107–2.300) and *HIF2B* rs12593988 (p = 0.024; HR = 0.690; 95% CI: 0.500–0.952) polymorphisms were associated with the risk of death in the univariate analysis (p<0.05) (**Table S8 in File S1**). In multivariable analysis, associations of *HIF2A* rs4953352 (p<0.001; HR = 2.189; 95% CI: 1.468–3.265) and *HIF2B* rs12593988 (p = 0.009; HR = 0.627; 95% CI: 0.442–0.890) with overall survival remained significant when also adjusted for vascular invasion status, sex, stage and MSI status (**Table 5**).

**Table 5:** Multivariable analysis results for overall survival (phase II; n = 477).

			95% CI for HR	
Variables	p-value	HR	(lower)	(upper)
*HIF2A* rs4953352 (TC+CC vs TT)	**<0.001**	2.189	1.468	3.265
*HIF2B* rs12593988 (GA+AA vs GG)	**0.009**	0.627	0.442	0.890
Sex (male vs female)	0.116	1.310	0.936	1.834
Stage	**<0.001**			
Stage (II vs I)	0.166	1.491	0.847	2.623
Stage (III vs I)	**0.039**	1.846	1.033	3.299
Stage (IV vs I)	**<0.001**	9.746	5.287	17.965
Vascular invasion (+ vs −)	0.179	1.257	0.901	1.755
MSI status (MSI-H vs MSS/MSI-L)	**0.002**	0.270	0.118	0.616

(+): present, (−): absent, CI: confidence interval, HR: hazard ratio, MSI-H: microsatellite instability-high, MSI-L: microsatellite instability-low, MSS: microsatellite stable. Significant associations (p<0.05) are shown in bold.

In univariate disease free survival analysis, *HIF2A* rs4953352 (p = 0.009; HR = 1.574; 95% CI: 1.122–2.207), *HIF2B* rs12593988 (p = 0.042; HR = 0.736; 95% CI: 0.548–0.988), and *HIF2B* rs8033706 (p = 0.023; HR = 0.704; 95% CI: 0.521–0.953) polymorphisms were associated with the risk of recurrence, metastasis or death (p<0.05) (**Table S9 in File S1**). In multivariable analysis, *HIF2A* rs4953352 (p<0.001; HR = 1.965; 95% CI: 1.366–2.828) and *HIF2B* rs12593988 (p = 0.017; HR = 0.678; 95% CI: 0.493–0.931) remained significantly associated with DFS time when adjusted for sex, location, stage, vascular invasion and MSI status (**Table 6**). Of note, since the genotype frequencies of the *HIF2B* rs8033706 polymorphism deviated from HWE, it was not included in this multivariable model.

**Table 6:** Multivariable analysis results for disease free survival (phase II, n = 476).

			95% CI for HR	
Variables	p-value	HR	(lower)	(upper)
*HIF2A* rs4953352 (TC+CC vs TT)	<0**.001**	1.965	1.366	2.828
*HIF2B* rs12593988 (GA+AA vs GG)	**0.017**	0.678	0.493	0.931
Sex (male vs female)	0.137	1.267	0.928	1.730
Location (rectum vs colon)	0.119	1.277	0.939	1.736
Stage	**<0.001**			
Stage (II vs I)	0.231	1.352	0.825	2.214
Stage (III vs I)	**0.030**	1.755	1.057	2.914
Stage (IV vs I)	**<0.001**	5.599	3.189	9.831
Vascular invasion (+ vs −)	0.261	1.196	0.876	1.633
MSI status (MSI-H vs MSS/MSI-L)	**0.018**	0.454	0.236	0.874

(+): present, (−): absent, CI: confidence interval, HR: hazard ratio, MSI-H: microsatellite instability-high, MSI-L: microsatellite instability-low, MSS: microsatellite stable. Significant associations (p<0.05) are shown in bold.

### SNPs investigated in both phase I and phase II cohorts

A total of 13 SNPs were investigated in both cohorts. In addition, according to the HapMap data, there were 15 polymorphisms investigated in phase I, whose genotypes were highly correlated (r^2^≥0.8) with 15 other polymorphisms investigated in phase II (**Table S3 in File S1**). We reasoned that the SNPs with highly correlated genotypes can serve as surrogates for each other. These proxy SNPs prompted us to check whether an association of a polymorphism detected in one cohort was replicated in the other cohort.

For the *HIF2A* rs11125070 polymorphism associated with disease free survival in the phase I cohort, the *HIF2A* rs4953342 polymorphism investigated in the phase II cohort was a proxy (r^2^>0.90). Our results showed that *HIF2A* rs4953342 was not associated with DFS in the phase II cohort (**Table S9 in File S1**). Additionally, for the *HIF2A* rs4953352 polymorphism that was detected to be associated with both overall and disease free survivals in the phase II cohort, there was a polymorphism (*HIF2A* rs6706003) genotyped in phase I with highly correlated genotypes (r^2^ = 0.87). Similarly, this polymorphism was not detected to be associated with either OS (**Table S5 in File S1**) or DFS (**Table S7 in File S1**) in the phase I cohort.

There was no proxy SNP studied in phase I cohort for the *HIF2B* rs12593988 polymorphism that we detected as associated with OS and DFS times in the patient cohort II.

### Differences between the phase I and phase II cohorts in terms of their clinicopathological features

Phase I and phase II cohorts significantly differed from each other in terms of the following baseline characteristics: age: p<0.001, sex: p = 0.037, grade: p<0.001, lymphatic invasion: p<0.001, location: p<0.001 and stage: p = 0.018.

## Discussion

In this study, we aimed to investigate the associations of genetic variations from select genes functioning in the hypoxia pathway and clinical outcome in colorectal cancer patients. This study involves two different cohorts and somehow overlapping yet not identical sets of genes and SNPs as depicted in **Figure 1**. Excluding the 13 SNPs that were common between phase I and II, a total of 113 different SNPs were investigated in either phase I or phase II.

In phase I of this project, 49 SNPs from six genes in hypoxia pathway and their relation to outcome in the patient cohort was analyzed using three different measures of outcome (OS, DFS and DSS). Our results showed that there was no association of these polymorphisms with OS or DSS in this cohort. However, one frequent SNP located in the mRNA coding region of *HIF2A* gene (rs11125070, NM_001430.4:c.27-21086A>T; minor allele frequency: 30.4% in phase I cohort) was associated with DFS in multivariable analysis independent of other prognostic indicators. Specifically, patients with the AT and TT genotypes (genotypes containing the minor allele T) had ∼0.4 times decreased risk of recurrence, metastasis or death compared to the patients with the AA genotype. However, when the association of a highly correlated polymorphism investigated in phase II cohort (*HIF2A* rs4953342) was tested in relation to disease free survival, this association was not detected in the phase II cohort. Therefore, while the differences between the two cohorts in terms of their clinicopathological features may have contributed to this discrepancy, considering the fact that no correction for multiple testing was applied in this study, we assume that the association observed in the phase I cohort was a false positive association.

In phase II of this project, out of the 77 SNPs investigated two SNPs (*HIF2B* rs12593988 and *HIF2A* rs4953352) were associated with outcome in both OS and DFS multivariable analyses. *HIF2B* rs12593988 (NM_014862.3:c.31+8939A>G) and *HIF2A* rs4953352 (NM_001430.4:c.27-8490T>C) are both frequent polymorphisms (minor allele frequencies 19% and 49%, respectively). Currently their biological consequences are unknown, however, a regulatory role of these variants in influencing the gene expression or function cannot be ruled out. For *HIF2A* rs4953352, another polymorphism with highly correlated genotypes (*HIF2A* rs6706003) was not associated with either OS or DFS in the phase I cohort. Non-replication of this association may be attributed to the differences between the two cohorts or to the small sample size of the phase I cohort which may lead to an insufficient study power to detect this association. However, if a correction for multiple testing procedure was applied, the observed association would not remain significant. Thus, the most likely explanation is that the association observed in the phase II cohort was a false-positive association.

There was no proxy SNP for the *HIF2B* rs12593988 in our cohort I dataset; hence we were not able to test its association with disease outcomes in an independent cohort.

Presently, studies testing the associations of the polymorphisms of the hypoxia genes with overall or disease free survivals in colorectal cancer are quite rare. For example, according to the dbCPCO database [17] and a literature search performed, as of January 2014 only four studies looked at the polymorphisms from the genes investigated in this study (*HIF1A*, *ARNT/HIF1B*, and *CXCL12*). The only study studied the *HIF1B* rs2228099 (Val174Val G/C) polymorphism did not find an association of it with overall survival in a patient cohort [18]. Additionally, two polymorphisms from the *HIF1A* gene (rs11549465 Pro582Ser C/T [18], [19] and rs11549467 Ala588Thr G/A [19] were investigated in relation to overall or disease free survivals in other cohorts: however these studies did not find an association of these polymorphisms with these outcomes in their patient cohorts. Finally, one *CXCL12* gene polymorphism, G/A in 3′-UTR (rs1801157 G801A), was examined in relation to disease free survival in two published studies. While in one study this polymorphism was not associated with disease free survival [20], in another study it was found to be associated with disease free survival in both univariate and multivariable analyses only in the patients without lymph node metastasis [21]. These literature findings show the rarity of published research including the polymorphisms from our list of genes. In addition, among these previously investigated polymorphisms, only the *HIF1B* rs2228099 polymorphism was investigated in our study (in both phase I and II). This indicates that except one polymorphism (*HIF1B* rs2228099), polymorphisms reported in this manuscript are investigated for the first time in relation to survival outcomes in colorectal cancer.

Our study has certain limitations and strengths. A) As this was an exploratory analysis, in order to minimize the false negative findings a correction for multiple testing was not performed. We should note that none of the associations detected in this study would remain significant after applying, for example, the Bonferroni test for correction. B) The patient cohort studied in phase I was characterized by a relatively small sample size (n = 272) and the phase I and II cohorts differed significantly from each other in terms of some baseline clinicopathological features. In addition, phase II patient cohort was inclined towards earlier stages and thus was not representative of the NFCCR cohort (data not shown). However, to our knowledge the phase II cohort (n = 535) is also one of the largest cohorts investigated in such a study in colorectal cancer. C) This study was limited with eight genes functioning in the hypoxia pathway (*HIF1A*, *HIF1B*, *HIF2A*, *HIF2B*, and *HIF3A*, *MIF*, *CXCL12*, and *LOX*); many other genes in this pathway were not investigated. D) Polymorphisms included in this study were selected based on the genotype correlation data, reducing the redundancy in experiments and statistical analysis, though we also acknowledge that possibly many other polymorphisms in these genes were missed and are thus candidates for further investigations.

In conclusion, this is a study that investigated a large number of polymorphisms from the hypoxia pathway genes, the majority of which were studied in relation to disease outcomes for the first time in colorectal cancer. Our results suggest that the *HIF2B* rs12593988 polymorphism may be associated with overall and disease free survivals in colorectal cancer, however, because of the large number of tests performed in this study, these associations are likely to be false-positives. We nevertheless report these associations for the researchers who may be interested in investigating *HIF2B* rs12593988 in relation to outcome in their future studies. Overall, our results point to no evidence of associations of the polymorphisms with the disease outcomes investigated in this study.

## Supporting Information

File S1 **Supporting information.** **Table S1,** HWE: Hardy-Weinberg equilibrium, MAF: minor allele frequency. **Table S2,** HWE: Hardy-Weinberg equilibrium, MAF: minor allele frequency. Numbers are rounded to the second decimal digit. **Table S5,** (+): present, (−): absent, CI: confidence interval, HR: hazard ratio, MSI-H: microsatellite instability-high, MSI-L: microsatellite instability-low, MSS: microsatellite stable, n: number of patients, OS: overall survival. Significant associations (p<0.05) are shown in bold. **Table S6,** (+): present (−): absent, CI: confidence interval, DSS: disease specific survival, HR: hazard ratio, MSI-H: microsatellite instability-high, MSI-L: microsatellite instability-low, MSS: microsatellite stable, n: number of patients. Significant associations (p<0.05) are shown in bold. **Table S7,** (+): present, (−): absent, CI: confidence interval, DFS: disease free survival, HR: hazard ratio, MSI-H: microsatellite instability-high, MSI-L: microsatellite instability-low, MSS: microsatellite stable, n: number of patients. Significant associations (p<0.05) are shown in bold. **Table S8,** (+): present, (−): absent, CI: confidence interval, HR: hazard ratio, MSI-H: microsatellite instability-high, MSI-L: microsatellite instability-low, MSS: microsatellite stable, n: number of patients, OS: overall survival. Significant associations (p<0.05) are shown in bold. **Table S9,** (+): present, (−): absent, CI: confidence interval, DFS: disease free survival, HR: hazard ratio, MSI-H: microsatellite instability-high, MSI-L: microsatellite instability-low, MSS: microsatellite stable, n: number of patients. Significant associations (p<0.05) are shown in bold.
(DOC)
