# Supplementary material for: Examining the Polymorphisms in the Hypoxia Pathway Genes in Relation to Outcome in Colorectal Cancer
Source: PLoS One. 2014 Nov 18;9(11):e113513. doi: 10.1371/journal.pone.0113513 (PMC4236175; doi:10.1371/journal.pone.0113513)
Supplement: File S1 — Supporting information. Table S1, HWE: Hardy-Weinberg equilibrium, MAF: minor allele frequency. Table S2, HWE: Hardy-Weinberg equilibrium, MAF: minor allele frequency. Numbers are rounded to the second decimal digit. Table S5, (+): present, (−): absent, CI: confidence interval, HR: hazard ratio, MSI-H: microsatellite instability-high, MSI-L: microsatellite instability-low, MSS: microsatellite stable, n: number of patients, OS: overall survival. Significant associations (p<0.05) are shown in bold. Table S6, (+): present (−): absent, CI: confidence interval, DSS: disease specific survival, HR: hazard ratio, MSI-H: microsatellite instability-high, MSI-L: microsatellite instability-low, MSS: microsatellite stable, n: number of patients. Significant associations (p<0.05) are shown in bold. Table S7, (+): present, (−): absent, CI: confidence interval, DFS: disease free survival, HR: hazard ratio, MSI-H: microsatellite instability-high, MSI-L: microsatellite instability-low, MSS: microsatellite stable, n: number of patients. Significant associations (p<0.05) are shown in bold. Table S8, (+): present, (−): absent, CI: confidence interval, HR: hazard ratio, MSI-H: microsatellite instability-high, MSI-L: microsatellite instability-low, MSS: microsatellite stable, n: number of patients, OS: overall survival. Significant associations (p<0.05) are shown in bold. Table S9, (+): present, (−): absent, CI: confidence interval, DFS: disease free survival, HR: hazard ratio, MSI-H: microsatellite instability-high, MSI-L: microsatellite instability-low, MSS: microsatellite stable, n: number of patients. Significant associations (p<0.05) are shown in bold. (DOC) [file pone.0113513.s001.doc]

**Table S1.** SNPs studied in phase I

| **SNP** | **Genotyping technique** | **Duplication rate (%)** | **Missing genotype (n)** | **Missing genotype (%)** | **Missing data >15%** | **MAF** | **Deviated from HWE** |
| --- | --- | --- | --- | --- | --- | --- | --- |
| *LOX* rs2956540 | MassArray | 5.2 | 9 | 3.21 |  | 0.461 |  |
| *LOX* rs2288393 | MassArray | 5.2 | 9 | 3.21 |  | 0.153 |  |
| *LOX* rs10040971 | MassArray | 5.2 | 8 | 2.86 |  | 0.145 | yes |
| *LOX* rs10519694 | MassArray | 5.2 | 8 | 2.86 |  | 0.285 |  |
| *HIF1A* rs2301106 | MassArray | 5.2 | 9 | 3.21 |  | 0.103 |  |
| *HIF1A* rs2301111 | MassArray | 5.2 | 8 | 2.86 |  | 0.165 |  |
| *HIF1A* rs2301113 | MassArray | 5.2 | 8 | 2.86 |  | 0.221 |  |
| *HIF1A* rs11158358 | MassArray | 5.2 | 8 | 2.86 |  | 0.123 |  |
| *HIF1B* rs2228099 | MassArray | 5.2 | 8 | 2.86 |  | 0.342 |  |
| *HIF1B* rs3738483 | MassArray | 5.2 | 8 | 2.86 |  | 0.096 |  |
| *HIF1B* rs11204737 | MassArray | 5.2 | 9 | 3.21 |  | 0.437 |  |
| *HIF1B* rs10847 | TaqMan® | 10.6 | 19 | 6.79 |  | 0.257 | yes |
| *CXCL12* rs2236534 | MassArray | 5.2 | 9 | 3.21 |  | 0.218 | yes |
| *CXCL12* rs2839688 | MassArray | 5.2 | 16 | 5.71 |  | 0.125 |  |
| *CXCL12* rs2236533 | TaqMan® | 7.6 | 8 | 2.86 |  | 0.297 | yes |
| *CXCL12* rs11592974 | TaqMan® | 37.83 | 8 | 2.86 |  | 0.230 | yes |
| *MIF* rs2096525 | MassArray | 5.2 | 17 | 6.07 |  | 0.167 |  |
| *HIF2A* rs2121266 | TaqMan® | 8.76 | 29 | 10.36 |  | 0.388 |  |
| *HIF2A* rs2346175 | TaqMan® | 12.97 | 65 | 23.21 | yes | 0.457 |  |
| *HIF2A* rs3768730 | TaqMan® | 9.31 | 33 | 11.79 |  | 0.474 |  |
| *HIF2A* rs4952818 | TaqMan® | 8.8 | 30 | 10.71 |  | 0.464 |  |
| *HIF2A* rs4953340 | TaqMan® | 12.9 | 32 | 11.43 |  | 0.381 |  |
| *HIF2A* rs6753127 | TaqMan® | 8.33 | 28 | 10 |  | 0.069 |  |
| *HIF2A* rs7583558 | TaqMan® | 11.98 | 38 | 13.57 |  | 0.312 |  |
| *HIF2A* rs9679290 | TaqMan® | 6.35 | 28 | 10 |  | 0.450 |  |
| *HIF2A* rs9973653 | TaqMan® | 13.22 | 38 | 13.57 |  | 0.306 | yes |
| *HIF2A* rs10178633 | TaqMan® | 12.4 | 40 | 14.29 |  | 0.419 |  |
| *HIF2A* rs11687512 | TaqMan® | 11.6 | 30 | 10.71 |  | 0.040 |  |
| *HIF2A* rs10199201 | MassArray | 5.2 | 30 | 10.71 |  | 0.154 |  |
| *HIF2A* rs11125070 | MassArray | 5.2 | 33 | 11.79 |  | 0.304 |  |
| *HIF2A* rs12614710 | MassArray | 5.2 | 32 | 11.43 |  | 0.440 |  |
| *HIF2A* rs13019414 | MassArray | 5.2 | 29 | 10.36 |  | 0.390 |  |
| *HIF2A* rs13412887 | MassArray | 5.2 | 31 | 11.07 |  | 0.187 |  |
| *HIF2A* rs1374748 | MassArray | 5.2 | 30 | 10.71 |  | 0.146 |  |
| *HIF2A* rs1562453 | MassArray | 5.2 | 32 | 11.43 |  | 0.492 |  |
| *HIF2A* rs1868084 | MassArray | 5.2 | 31 | 11.07 |  | 0.213 |  |
| *HIF2A* rs1868087 | MassArray | 5.2 | 30 | 10.71 |  | 0.182 |  |
| *HIF2A* rs1992846 | MassArray | 5.2 | 31 | 11.07 |  | 0.211 |  |
| *HIF2A* rs2034327 | MassArray | 5.2 | 37 | 13.21 |  | 0.498 |  |
| *HIF2A* rs2044456 | MassArray | 5.2 | 41 | 14.64 |  | 0.335 |  |
| *HIF2A* rs2346176 | MassArray | 5.2 | 29 | 10.36 |  | 0.373 |  |
| *HIF2A* rs3768728 | MassArray | 5.2 | 29 | 10.36 |  | 0.131 |  |
| *HIF2A* rs4145836 | MassArray | 5.2 | 29 | 10.36 |  | 0.124 | yes |
| *HIF2A* rs4953344 | MassArray | 5.2 | 32 | 11.43 |  | 0.135 |  |
| *HIF2A* rs4953349 | MassArray | 5.2 | 35 | 12.5 |  | 0.488 |  |
| *HIF2A* rs4953353 | MassArray | 5.2 | 29 | 10.36 |  | 0.339 |  |
| *HIF2A* rs6706003 | MassArray | 5.2 | 30 | 10.71 |  | 0.496 |  |
| *HIF2A* rs6712143 | MassArray | 5.2 | 29 | 10.36 |  | 0.263 |  |
| *HIF2A* rs7583392 | MassArray | 5.2 | 29 | 10.36 |  | 0.470 |  |

**Table S2.** SNPs studied in phase II

| **Gene** | **SNP ID** | **Missing genotype (n)** | **Missing genotype (%)** | **Missing data >15%** | **MAF** | ***Deviated from HWE** |
| --- | --- | --- | --- | --- | --- | --- |
| *HIF1A* | rs2301106 | 0 | 0 |  | 0.13 |  |
| *HIF1A* | rs12434438 | 0 | 0 |  | 0.22 |  |
| *HIF1A* | rs2057482 | 0 | 0 |  | 0.15 |  |
| *HIF1B* | rs10847 | 0 | 0 |  | 0.27 |  |
| *HIF1B* | rs2228099 | 0 | 0 |  | 0.34 |  |
| *HIF1B* | rs11204737 | 0 | 0 |  | 0.41 |  |
| *HIF2A* | rs1867785 | 0 | 0 |  | 0.36 |  |
| *HIF2A* | rs2121266 | 1 | 0.19 |  | 0.38 |  |
| *HIF2A* | rs17034950 | 0 | 0 |  | 0.26 |  |
| *HIF2A* | rs9973653 | 0 | 0 |  | 0.28 |  |
| *HIF2A* | rs4953342 | 0 | 0 |  | 0.3 |  |
| *HIF2A* | rs1868089 | 0 | 0 |  | 0.49 |  |
| *HIF2A* | rs6758592 | 0 | 0 |  | 0.44 |  |
| *HIF2A* | rs12614710 | 0 | 0 |  | 0.47 |  |
| *HIF2A* | rs4953352 | 0 | 0 |  | 0.49 |  |
| *HIF2A* | rs4953353 | 0 | 0 |  | 0.34 |  |
| *HIF2A* | rs2346175 | 0 | 0 |  | 0.46 |  |
| *HIF2A* | rs6756667 | 0 | 0 |  | 0.48 |  |
| *HIF2A* | rs1868086 | 1 | 0.19 |  | 0.2 |  |
| *HIF2A* | rs6712143 | 0 | 0 |  | 0.27 |  |
| *HIF2A* | rs10176396 | 0 | 0 |  | 0.31 |  |
| *HIF2A* | rs1374748 | 0 | 0 |  | 0.13 |  |
| *HIF2A* | rs3768728 | 0 | 0 |  | 0.14 |  |
| *HIF2A* | rs2346176 | 0 | 0 |  | 0.38 |  |
| *HIF2A* | rs10178633 | 0 | 0 |  | 0.44 |  |
| *HIF2A* | rs7594912 | 0 | 0 |  | 0.45 |  |
| *HIF2A* | rs7557402 | 0 | 0 |  | 0.46 |  |
| *HIF2A* | rs7571218 | 0 | 0 |  | 0.38 |  |
| *HIF2B* | rs12591286 | 0 | 0 |  | 0.35 |  |
| *HIF2B* | rs8041826 | 0 | 0 |  | 0.17 | yes |
| *HIF2B* | rs1446337 | 0 | 0 |  | 0.25 |  |
| *HIF2B* | rs12593988 | 0 | 0 |  | 0.19 |  |
| *HIF2B* | rs3848206 | 1 | 0.19 |  | 0.5 |  |
| *HIF2B* | rs3848207 | 0 | 0 |  | 0.14 |  |
| *HIF2B* | rs7172914 | 0 | 0 |  | 0.21 | yes |
| *HIF2B* | rs10431813 | 2 | 0.37 |  | 0.26 |  |
| *HIF2B* | rs3910982 | 0 | 0 |  | 0.31 |  |
| *HIF2B* | rs11635014 | 0 | 0 |  | 0.41 |  |
| *HIF2B* | rs1020398 | 0 | 0 |  | 0.27 | yes |
| *HIF2B* | rs11633642 | 0 | 0 |  | 0.34 |  |
| *HIF2B* | rs7184010 | 1 | 0.19 |  | 0.26 |  |
| *HIF2B* | rs3848170 | 0 | 0 |  | 0.11 |  |
| *HIF2B* | rs4778791 | 0 | 0 |  | 0.48 |  |
| *HIF2B* | rs8034535 | 0 | 0 |  | 0.15 |  |
| *HIF2B* | rs895442 | 0 | 0 |  | 0.11 |  |
| *HIF2B* | rs1037124 | 0 | 0 |  | 0.16 |  |
| *HIF2B* | rs1374213 | 1 | 0.19 |  | 0.44 |  |
| *HIF2B* | rs3901896 | 0 | 0 |  | 0.4 |  |
| *HIF2B* | rs2278709 | 0 | 0 |  | 0.29 |  |
| *HIF2B* | rs8028295 | 0 | 0 |  | 0.45 |  |
| *HIF2B* | rs4609803 | 0 | 0 |  | 0.27 |  |
| *HIF2B* | rs4778800 | 0 | 0 |  | 0.22 |  |
| *HIF2B* | rs7178902 | 0 | 0 |  | 0.44 |  |
| *HIF2B* | rs4238521 | 0 | 0 |  | 0.15 |  |
| *HIF2B* | rs4331301 | 0 | 0 |  | 0.38 |  |
| *HIF2B* | rs4778600 | 1 | 0.19 |  | 0.15 | yes |
| *HIF2B* | rs11856676 | 0 | 0 |  | 0.46 |  |
| *HIF2B* | rs4238522 | 0 | 0 |  | 0.4 |  |
| *HIF2B* | rs4074666 | 0 | 0 |  | 0.47 |  |
| *HIF2B* | rs11635554 | 0 | 0 |  | 0.28 |  |
| *HIF2B* | rs4778818 | 0 | 0 |  | 0.26 |  |
| *HIF2B* | rs4778819 | 0 | 0 |  | 0.29 |  |
| *HIF2B* | rs7403706 | 0 | 0 |  | 0.2 |  |
| *HIF2B* | rs6495509 | 0 | 0 |  | 0.21 |  |
| *HIF2B* | rs8039725 | 0 | 0 |  | 0.19 |  |
| *HIF2B* | rs8033706 | 56 | 10.49 |  | 0.49 | yes |
| *HIF2B* | rs4301984 | 0 | 0 |  | 0.22 |  |
| *HIF2B* | rs4459508 | 0 | 0 |  | 0.28 |  |
| *HIF3A* | rs2072491 | 0 | 0 |  | 0.11 |  |
| *HIF3A* | rs757638 | 0 | 0 |  | 0.15 |  |
| *HIF3A* | rs12461322 | 0 | 0 |  | 0.11 | yes |
| *HIF3A* | rs887946 | 0 | 0 |  | 0.32 |  |
| *HIF3A* | rs3764610 | 0 | 0 |  | 0.17 |  |
| *HIF3A* | rs11665853 | 0 | 0 |  | 0.11 | yes |
| *LOX* | rs3792802 | 1 | 0.19 |  | 0.17 |  |
| *LOX* | rs1800449 | 0 | 0 |  | 0.17 |  |
| *CXCL12* | rs2839695 | 0 | 0 |  | 0.2 |  |

**Table S3.** SNPs that are common or with highly correlated genotypes investigated in both phase I and phase II

| **phase I SNPs** | **phase I SNPs analyzed in phase II** | **SNP investigated in phase II that are highly correlated with SNPs in phase I** | **r^2^** |
| --- | --- | --- | --- |
| *LOX* rs10040971 |  | rs3792802 | 0.831 |
| *LOX* rs2288393 |  | rs1800449 | 0.944 |
| *HIF1A* rs2301106 | rs2301106 |  |  |
| *HIF1A* rs2301111 |  | rs12434438 | 1 |
| *HIF1A* rs11158358 |  | rs2057482 | 0.929 |
| *HIF1B* rs2228099 | rs2228099 |  |  |
| *HIF1B* rs11204737 | rs11204737 |  |  |
| *HIF1B* rs10847 | rs10847 |  |  |
| *CXCL12* rs11592974 |  | rs2839695 | 1 |
| *HIF2A* rs2121266 | rs2121266 |  |  |
| *HIF2A* rs2346175 | rs2346175 |  |  |
| *HIF2A* rs3768730 |  | rs7594912 | 0.871 |
| *HIF2A* rs9679290 |  | rs6758592 | 0.967 |
| *HIF2A* rs9973653 | rs9973653 |  |  |
| *HIF2A* rs10178633 | rs10178633 |  |  |
| *HIF2A* rs11125070 |  | rs4953342 | 0.913 |
| *HIF2A* rs12614710 | rs12614710 |  |  |
| *HIF2A* rs13019414 |  | rs7571218 | 0.933 |
| *HIF2A* rs1562453 |  | rs6756667 | 0.967 |
| *HIF2A* rs1868087 |  | rs1868086 | 1 |
| *HIF2A* rs2044456 |  | rs17034950 | 0.921 |
| *HIF2A* rs2346176 | rs2346176 |  |  |
| *HIF2A* rs3768728 | rs3768728 |  |  |
| *HIF2A* rs4953349 |  | rs1868089 | 1 |
| *HIF2A* rs4953353 | rs4953353 |  |  |
| *HIF2A* rs6706003 |  | rs4953352 | 0.87 |
| *HIF2A* rs6712143 | rs6712143 |  |  |
| *HIF2A* rs7583392 |  | rs7557402 | 0.964 |

**Table S4.** Assay IDs for 14 polymorphisms genotyped using the Applied Biosystems’ TaqMan® SNP genotyping assays

| **SNP ID** | **Assay ID** |
| --- | --- |
| rs9679290 | C_229862_10 |
| rs4952818 | C_2148920_20 |
| rs2346175 | C_11158065_10 |
| rs6753127 | C_29117756_10 |
| rs7583554 | C_29117759_10 |
| rs9973653 | C_30621213_10 |
| rs3768730 | C_2162965_10 |
| rs11687512 | C_2148916_10 |
| rs10178633 | C_2163036_10 |
| rs2236533 | C_15954640_10 |
| rs11592974 | C_17776531_10 |
| rs10847 | AHHSOC5 |
| rs2121266 | C_16104055_20 |
| rs4953340 | C_29557501_10 |

**Table S5.** Univariate analysis results for OS (phase I)

| **Variables** | **p-value** | **HR** | **95% CI** | | **n** |
| --- | --- | --- | --- | --- | --- |
|  |  |  | **Lower** | **Upper** |  |
| *LOX* rs2956540 (GC+GG vs CC) | .571 | .910 | .656 | 1.262 | 271 |
| *LOX* rs2288393 (CG+CC vs GG) | .946 | .988 | .704 | 1.387 | 271 |
| *LOX* rs10040971 (CT+CC vs TT) | .854 | 1.033 | .733 | 1.456 | 272 |
| *LOX* rs10519694 (CT +TT vs CC) | **.046** | .735 | .543 | .994 | 272 |
| *HIF1A* rs2301106 (TC + CC vs TT) | .247 | .787 | .524 | 1.180 | 271 |
| *HIF1A* rs2301111 (GC+GG vs CC) | .230 | .813 | .579 | 1.140 | 272 |
| *HIF1A* rs2301113 (CA +CC vs AA) | .721 | .945 | .693 | 1.289 | 272 |
| *HIF1A* rs11158358 (GC+GG vs CC) | .106 | .731 | .499 | 1.068 | 272 |
| *HIF1B* rs2228099 (GC +CC vs GG) | .506 | .902 | .666 | 1.222 | 272 |
| *HIF1B* rs3738483 (AG +AA vs GG) | .241 | 1.255 | .858 | 1.836 | 272 |
| *HIF1B* rs11204737 (CT+CC vs TT) | .250 | 1.220 | .869 | 1.713 | 271 |
| *HIF1B* rs10847 (AG +AA vs GG) | .775 | 1.046 | .767 | 1.427 | 261 |
| *CXCL12* rs2236534 (GT+TT vs GG) | .563 | 1.097 | .801 | 1.504 | 271 |
| *CXCL12* rs2839688 (CG+CC vs GG) | .741 | 1.064 | .738 | 1.534 | 264 |
| *CXCL12* rs2236533 (GA +AA vs GG) | .690 | 1.065 | .780 | 1.455 | 261 |
| *CXCL12* rs11592974 (TC+CC vs TT) | .531 | .904 | .661 | 1.238 | 267 |
| *MIF* rs2096525 (TC+CC vs TT) | .770 | .951 | .681 | 1.329 | 263 |
| *HIF2A* rs2121266 (AC +CC vs AA) | .312 | 1.191 | .849 | 1.672 | 251 |
| *HIF2A* rs2346175 (CT +CC vs TT) | .565 | .889 | .595 | 1.329 | 185 |
| *HIF2A* rs3768730 (GT +TT vs GG) | .920 | 1.018 | .713 | 1.454 | 247 |
| *HIF2A* rs4952818 (TC +CC vs TT) | .941 | .987 | .692 | 1.407 | 250 |
| *HIF2A* rs4953340 (GC +CC vs GG) | .722 | .942 | .677 | 1.310 | 248 |
| *HIF2A* rs6753127 (CT +TT vs CC) | .208 | .729 | .446 | 1.193 | 252 |
| *HIF2A* rs7583558 (CT +CC vs TT) | .324 | .850 | .615 | 1.174 | 242 |
| *HIF2A* rs9679290 (CG +CC vs GG) | .137 | .777 | .557 | 1.083 | 252 |
| *HIF2A* rs9973653 (GT +TT vs GG) | .731 | .945 | .684 | 1.306 | 242 |
| *HIF2A* rs10178633 (GA +AA vs GG) | .860 | 1.031 | .736 | 1.444 | 240 |
| *HIF2A* rs11687512 (GC+CC vs GG ) | .252 | .688 | .362 | 1.306 | 250 |
| *HIF2A* rs10199201 (CT + CC vs TT) | .849 | .967 | .681 | 1.371 | 250 |
| *HIF2A* rs11125070 (AT+TT vs AA) | **.003** | .616 | .447 | .848 | 247 |
| *HIF2A* rs12614710 (GT +TT vs GG) | .532 | 1.115 | .793 | 1.566 | 248 |
| *HIF2A* rs13019414 (GC +CC vs GG) | .254 | .830 | .603 | 1.143 | 251 |
| *HIF2A* rs13412887 (CG+GG vs CC) | .101 | .752 | .534 | 1.057 | 249 |
| *HIF2A* rs1374748 (GT +TT vs GG) | .563 | 1.110 | .780 | 1.578 | 250 |
| *HIF2A* rs1562453 (CT +CC vs TT) | .919 | 1.019 | .714 | 1.452 | 248 |
| *HIF2A* rs1868084 (GC+GG vs CC) | **.024** | .678 | .483 | .950 | 249 |
| *HIF2A* rs1868087 (AG+GG vs AA) | .823 | .962 | .688 | 1.346 | 250 |
| *HIF2A* rs1992846 (CT +TT vs CC) | .424 | 1.141 | .826 | 1.578 | 249 |
| *HIF2A* rs2034327 (GC + CC vs GG) | .875 | .971 | .676 | 1.396 | 243 |
| *HIF2A* rs2044456 (GA+GG vs AA) | .612 | .919 | .663 | 1.273 | 239 |
| *HIF2A* rs2346176 (TC + CC vs TT) | .304 | 1.185 | .857 | 1.637 | 251 |
| *HIF2A* rs3768728 (TC +CC vs TT) | .398 | 1.166 | .816 | 1.667 | 251 |
| *HIF2A* rs4145836 (AG +AA vs GG) | .414 | .855 | .586 | 1.246 | 251 |
| *HIF2A* rs4953344 (CT +CC vs TT) | .061 | 1.391 | .985 | 1.965 | 248 |
| *HIF2A* rs4953349 (GT +TT vs GG) | .856 | 1.033 | .726 | 1.471 | 245 |
| *HIF2A* rs4953353 (GT +TT vs GG) | .402 | .874 | .637 | 1.198 | 251 |
| *HIF2A* rs6706003 (CG + CC vs GG) | .899 | 1.023 | .721 | 1.451 | 250 |
| *HIF2A* rs6712143 (GA+GG vs AA) | .948 | .990 | .722 | 1.357 | 251 |
| *HIF2A* rs7583392 (AG +AA vs GG) | .766 | .950 | .676 | 1.335 | 251 |
| Sex (male vs female) | .094 | 1.296 | .957 | 1.757 | 280 |
| Age | **<0.001** | 1.034 | 1.021 | 1.047 | 280 |
| Grade (poorly differentiated/ undifferentiated vs well/moderately differentiated) | **.001** | 1.949 | 1.325 | 2.866 | 276 |
| Histology (mucinous vs non-mucinous) | .603 | 1.113 | .743 | 1.669 | 280 |
| Lymphatic invasion (+ vs -) | **<0.001** | 2.472 | 1.648 | 3.710 | 180 |
| Location (rectum vs colon) | .343 | 1.191 | .830 | 1.708 | 280 |
| Stage | **<0.001** |  |  |  | 271 |
| Stage (II vs I) | .150 | 1.458 | .873 | 2.435 |  |
| Stage (III vs I) | **<0.001** | 2.464 | 1.483 | 4.094 |  |
| Stage (IV vs I) | **<0.001** | 9.647 | 5.629 | 16.534 |  |
| MSI status ( MSI-H vs MSS/MSI-L) | **.001** | .359 | .195 | .662 | 280 |

**Table S6.** Univariate analysis results for DSS (phase I)

| **Variables** | **p-value** | **HR** | **95% CI** | | **n** |
| --- | --- | --- | --- | --- | --- |
|  |  |  | **Lower** | **Upper** |  |
| *LOX* rs2956540 (GC+GG vs CC) | .649 | .911 | .611 | 1.359 | 271 |
| *LOX* rs2288393 ( CG + CC vs GG) | .995 | .999 | .657 | 1.519 | 271 |
| *LOX* rs10040971 (CT+CC vs TT) | .827 | 1.048 | .687 | 1.600 | 272 |
| *LOX* rs10519694 ( CT +TT vs CC) | .284 | .816 | .563 | 1.183 | 272 |
| *HIF1A* rs2301106 (TC + CC vs TT) | .092 | .626 | .364 | 1.080 | 271 |
| *HIF1A* rs2301111 (GC+GG vs CC) | .315 | .807 | .531 | 1.226 | 272 |
| *HIF1A* rs2301113 (CA +CC vs AA) | .377 | 1.184 | .814 | 1.721 | 272 |
| *HIF1A* rs11158358 (GC+GG vs CC) | .099 | .665 | .410 | 1.079 | 272 |
| *HIF1B* rs2228099 (GC + CC vs GG) | .279 | .814 | .561 | 1.181 | 272 |
| *HIF1B* rs3738483 (AG +AA vs GG) | .241 | 1.316 | .832 | 2.082 | 272 |
| *HIF1B* rs11204737 ( CT + CC vs TT) | .355 | 1.218 | .802 | 1.852 | 271 |
| *HIF1B* rs10847 (AG +AA vs GG) | .800 | 1.050 | .719 | 1.534 | 261 |
| *CXCL12* rs2236534 (GT+TT vs GG) | .231 | 1.263 | .862 | 1.850 | 271 |
| *CXCL12* rs2839688 ( CG + CC vs GG) | .949 | .985 | .622 | 1.561 | 264 |
| *CXCL12* rs2236533 (GA +AA vs GG) | .493 | 1.142 | .781 | 1.672 | 261 |
| *CXCL12* rs11592974 (TC + CC vs TT) | .110 | .722 | .485 | 1.077 | 267 |
| *MIF* rs2096525 (TC + CC vs TT) | .962 | .990 | .656 | 1.493 | 263 |
| *HIF2A* rs2121266 (AC +CC vs AA) | .711 | .927 | .620 | 1.385 | 251 |
| *HIF2A* rs2346175 (CT + CC vs TT) | .275 | .767 | .476 | 1.235 | 185 |
| *HIF2A* rs3768730 (GT +TT vs GG) | .638 | .902 | .588 | 1.385 | 247 |
| *HIF2A* rs4952818 (TC + CC vs TT) | .226 | .773 | .510 | 1.172 | 250 |
| *HIF2A* rs4953340 (GC + CC vs GG) | .218 | .780 | .525 | 1.158 | 248 |
| *HIF2A* rs6753127 (CT +TT vs CC) | .743 | .910 | .518 | 1.600 | 252 |
| *HIF2A* rs7583558 (CT +CC vs TT) | .127 | .735 | .495 | 1.092 | 242 |
| *HIF2A* rs9679290 (CG +CC vs GG) | .275 | .796 | .529 | 1.198 | 252 |
| *HIF2A* rs9973653 (GT +TT vs GG) | .092 | .712 | .480 | 1.057 | 242 |
| *HIF2A* rs10178633 (GA +AA vs GG) | .689 | .920 | .612 | 1.383 | 240 |
| *HIF2A* rs11687512 (GC+CC vs GG ) | .687 | .862 | .419 | 1.775 | 250 |
| *HIF2A* rs10199201 (CT + CC vs TT) | .737 | .928 | .600 | 1.435 | 250 |
| *HIF2A* rs11125070 (AT+TT vs AA) | .128 | .737 | .497 | 1.092 | 247 |
| *HIF2A* rs12614710 (GT +TT vs GG) | .724 | 1.078 | .711 | 1.634 | 248 |
| *HIF2A* rs13019414 (GC +CC vs GG) | .153 | .752 | .508 | 1.111 | 251 |
| *HIF2A* rs13412887 (CG+GG vs CC) | .486 | .864 | .572 | 1.304 | 249 |
| *HIF2A* rs1374748 (GT +TT vs GG) | .574 | 1.132 | .735 | 1.742 | 250 |
| *HIF2A* rs1562453 (CT + CC vs TT) | .675 | .913 | .596 | 1.398 | 248 |
| *HIF2A* rs1868084 (GC+GG vs CC) | .392 | .838 | .559 | 1.257 | 249 |
| *HIF2A* rs1868087 (AG+GG vs AA) | .927 | 1.019 | .677 | 1.534 | 250 |
| *HIF2A* rs1992846 (CT +TT vs CC) | .184 | .751 | .492 | 1.146 | 249 |
| *HIF2A* rs2034327 (GC + CC vs GG) | .768 | .935 | .600 | 1.458 | 243 |
| *HIF2A* rs2044456 (GA+GG vs AA) | .088 | .707 | .475 | 1.053 | 239 |
| *HIF2A* rs2346176 (TC + CC vs TT) | .896 | .974 | .658 | 1.442 | 251 |
| *HIF2A* rs3768728 (TC + CC vs TT) | .543 | 1.146 | .738 | 1.780 | 251 |
| *HIF2A* rs4145836 (AG +AA vs GG) | .940 | 1.017 | .652 | 1.588 | 251 |
| *HIF2A* rs4953344 (CT + CC vs TT) | .836 | 1.049 | .667 | 1.649 | 248 |
| *HIF2A* rs4953349 (GT +TT vs GG) | .946 | 1.015 | .660 | 1.562 | 245 |
| *HIF2A* rs4953353 (GT +TT vs GG) | .381 | .840 | .570 | 1.240 | 251 |
| *HIF2A* rs6706003 (CG + CC vs GG) | .918 | 1.023 | .665 | 1.573 | 250 |
| *HIF2A* rs6712143 (GA+GG vs AA) | .287 | .807 | .544 | 1.198 | 251 |
| *HIF2A* rs7583392 (AG +AA vs GG) | .356 | .825 | .548 | 1.242 | 251 |
| Sex (male vs female) | .246 | 1.248 | .858 | 1.814 | 280 |
| Age | **.029** | 1.017 | 1.002 | 1.032 | 280 |
| Grade (poorly differentiated/undifferentiated vs well/moderately differentiated) | **<0.001** | 2.333 | 1.493 | 3.647 | 276 |
| Histology (mucinous vs non-mucinous) | .774 | .926 | .545 | 1.571 | 280 |
| Lymphatic invasion (+ vs -) | **<0.001** | 3.017 | 1.821 | 4.998 | 180 |
| Location (rectum vs colon) | .520 | 1.157 | .742 | 1.805 | 280 |
| Stage | **<0.001** |  |  |  | 271 |
| Stage (II vs I) | .067 | 2.324 | .942 | 5.731 |  |
| Stage (III vs I) | **<0.001** | 6.067 | 2.562 | 14.36 |  |
| Stage (IV vs I) | **<0.001** | 28.082 | 11.784 | 66.92 |  |
| MSI status ( MSI-H vs MSS/MSI-L) | **.002** | .203 | .075 | .551 | 280 |

**Table S7.** Univariate analysis results for DFS (phase I)

| **Variables** | **p-value** | **HR** | **95% CI** | | **n** |
| --- | --- | --- | --- | --- | --- |
|  |  |  | **Lower** | **Upper** |  |
| *LOX* rs2956540 (GC + GG vs CC) | .225 | .824 | .603 | 1.126 | 271 |
| *LOX* rs2288393 ( CG + CC vs GG) | .982 | 1.004 | .725 | 1.390 | 271 |
| *LOX* rs10040971 (CT +CC vs TT) | .995 | 1.001 | .718 | 1.395 | 272 |
| *LOX* rs10519694 ( CT + TT vs CC) | **.012** | .685 | .510 | .919 | 272 |
| *HIF1A* rs2301106 (TC + CC vs TT) | .588 | .902 | .621 | 1.310 | 271 |
| *HIF1A* rs2301111 (GC+ GG vs CC) | .334 | .853 | .618 | 1.177 | 272 |
| *HIF1A* rs2301113 (CA + CC vs AA) | .646 | .932 | .691 | 1.258 | 272 |
| *HIF1A* rs11158358 (GC+ GG vs CC) | .291 | .826 | .580 | 1.177 | 272 |
| *HIF1B* rs2228099 (GC + CC vs GG) | .402 | .882 | .658 | 1.183 | 272 |
| *HIF1B* rs3738483 (AG + AA vs GG) | .253 | 1.240 | .857 | 1.794 | 272 |
| *HIF1B* rs11204737 ( CT + CC vs TT) | .261 | 1.206 | .870 | 1.671 | 271 |
| *HIF1B* rs10847 (AG + AA vs GG) | .847 | 1.030 | .763 | 1.391 | 261 |
| *CXCL12* rs2236534 (GT + TT vs GG) | .553 | 1.096 | .809 | 1.485 | 271 |
| *CXCL12* rs2839688 ( CG + CC vs GG) | .776 | .949 | .663 | 1.359 | 264 |
| *CXCL12* rs2236533 (GA +AA vs GG) | .520 | 1.104 | .817 | 1.490 | 261 |
| *CXCL12* rs11592974 (TC + CC vs TT) | .800 | .962 | .711 | 1.301 | 267 |
| *MIF* rs2096525 (TC + CC vs TT) | .339 | .852 | .613 | 1.183 | 263 |
| *HIF2A* rs2121266 (AC + CC vs AA) | .303 | 1.187 | .857 | 1.645 | 251 |
| *HIF2A* rs2346175 (CT + CC vs TT) | .989 | 1.003 | .679 | 1.481 | 185 |
| *HIF2A* rs3768730 (GT + TT vs GG) | .931 | .985 | .699 | 1.389 | 247 |
| *HIF2A* rs4952818 (TC + CC vs TT) | .960 | 1.009 | .716 | 1.421 | 250 |
| *HIF2A* rs4953340 (GC + CC vs GG) | .910 | .982 | .714 | 1.349 | 248 |
| *HIF2A* rs6753127 (CT + TT vs CC) | .321 | .793 | .502 | 1.254 | 252 |
| *HIF2A* rs7583558 (CT + CC vs TT) | .503 | .899 | .660 | 1.226 | 242 |
| *HIF2A* rs9679290 (CG + CC vs GG) | .123 | .777 | .564 | 1.071 | 252 |
| *HIF2A* rs9973653 (GT + TT vs GG) | .908 | 1.018 | .746 | 1.391 | 242 |
| *HIF2A* rs10178633 (GA + AA vs GG) | .835 | 1.035 | .747 | 1.434 | 240 |
| *HIF2A* rs11687512 (GC+ CC vs GG ) | .184 | .648 | .342 | 1.229 | 250 |
| *HIF2A* rs10199201 (CT + CC vs TT) | .734 | .943 | .674 | 1.320 | 250 |
| *HIF2A* rs11125070 (AT + TT vs AA) | **.003** | .629 | .461 | .858 | 247 |
| *HIF2A* rs12614710 (GT + TT vs GG) | .378 | 1.160 | .834 | 1.612 | 248 |
| *HIF2A* rs13019414 (GC + CC vs GG) | .318 | .854 | .627 | 1.164 | 251 |
| *HIF2A* rs13412887 (CG + GG vs CC) | .141 | .782 | .564 | 1.085 | 249 |
| *HIF2A* rs1374748 (GT + TT vs GG) | .842 | 1.035 | .735 | 1.458 | 250 |
| *HIF2A* rs1562453 (CT + CC vs TT) | .825 | .962 | .683 | 1.356 | 248 |
| *HIF2A* rs1868084 (GC + GG vs CC) | .122 | .776 | .563 | 1.070 | 249 |
| *HIF2A* rs1868087 (AG + GG vs AA) | .638 | .925 | .670 | 1.278 | 250 |
| *HIF2A* rs1992846 (CT + TT vs CC) | .541 | 1.102 | .807 | 1.505 | 249 |
| *HIF2A* rs2034327 (GC + CC vs GG) | .578 | .906 | .640 | 1.282 | 243 |
| *HIF2A* rs2044456 (GA + GG vs AA) | .691 | .938 | .684 | 1.286 | 239 |
| *HIF2A* rs2346176 (TC + CC vs TT) | .422 | 1.136 | .832 | 1.551 | 251 |
| *HIF2A* rs3768728 (TC + CC vs TT) | .654 | 1.083 | .765 | 1.532 | 251 |
| *HIF2A* rs4145836 (AG +AA vs GG) | .566 | .900 | .629 | 1.289 | 251 |
| *HIF2A* rs4953344 (CT + CC vs TT) | .257 | 1.218 | .866 | 1.713 | 248 |
| *HIF2A* rs4953349 (GT + TT vs GG) | .579 | 1.102 | .782 | 1.554 | 245 |
| *HIF2A* rs4953353 (GT + TT vs GG) | .486 | .897 | .662 | 1.217 | 251 |
| *HIF2A* rs6706003 (CG + CC vs GG) | .832 | 1.037 | .740 | 1.454 | 250 |
| *HIF2A* rs6712143 (GA+ GG vs AA) | .935 | .987 | .729 | 1.338 | 251 |
| *HIF2A* rs7583392 (AG + AA vs GG) | .460 | .884 | .637 | 1.227 | 251 |
| Sex (male vs female) | .230 | 1.196 | .893 | 1.601 | 280 |
| Age | **<0.001** | 1.025 | 1.012 | 1.037 | 280 |
| Grade (poorly differentiated/undifferentiated vs well/moderately differentiated) | **.008** | 1.666 | 1.141 | 2.432 | 276 |
| Histology (mucinous vs non-mucinous) | .661 | 1.092 | .738 | 1.614 | 280 |
| Lymphatic invasion (+ vs -) | **<0.001** | 2.077 | 1.414 | 3.051 | 180 |
| Location (rectum vs colon) | .273 | 1.213 | .859 | 1.711 | 280 |
| Stage | **<0.001** |  |  |  | 271 |
| Stage (II vs I) | **.040** | 1.695 | 1.024 | 2.806 |  |
| Stage (III vs I) | **<0.001** | 2.877 | 1.737 | 4.763 |  |
| Stage (IV vs I) | **<0.001** | 106.420 | 48.630 | 232.88 |  |
| MSI status (MSI-H vs MSS/MSI-L) | **<0.001** | .351 | .195 | .630 | 280 |

**Table S8.** Univariate analysis results for OS (phase II)

| **Variables** | **p-value** | **HR** | **95% CI for HR** | | **n** |
| --- | --- | --- | --- | --- | --- |
|  |  |  | **Lower** | **Upper** |  |
| *HIF1B* rs10847 (AG+AA vs GG) | .393 | .880 | .657 | 1.179 | 534 |
| *HIF1B* rs2228099 (GC+CC vs GG) | .689 | 1.062 | .791 | 1.427 | 534 |
| *HIF1B* rs11204737 (TC+CC vs TT) | .512 | 1.109 | .814 | 1.510 | 534 |
| *HIF2A* rs1867785 (AG+AA vs GG) | .847 | .971 | .722 | 1.306 | 534 |
| *HIF2A* rs2121266 (AC+CC vs AA) | .375 | .875 | .651 | 1.176 | 533 |
| *HIF2A* rs17034950 (AG+AA vs GG) | .809 | .965 | .720 | 1.292 | 534 |
| *HIF2A* rs9973653 (TG+TT vs GG) | .822 | .967 | .723 | 1.294 | 534 |
| *HIF2A* rs4953342 (AG+GG vs AA) | .542 | 1.095 | .818 | 1.464 | 534 |
| *HIF2A* rs1868089 (TC+CC vs TT) | .574 | .909 | .652 | 1.268 | 534 |
| *HIF2A* rs6758592 (TC+CC vs TT) | .259 | 1.203 | .873 | 1.659 | 534 |
| *HIF2A* rs12614710 (TG+TT vs GG) | .821 | 1.039 | .747 | 1.445 | 534 |
| *HIF2A* rs4953352 (TC+CC vs TT) | **.012** | 1.596 | 1.107 | 2.300 | 534 |
| *HIF2A* rs4953353 (TG+TT vs GG) | .195 | 1.217 | .904 | 1.639 | 534 |
| *HIF2A* rs2346175 (TC+CC vs TT) | .142 | 1.281 | .921 | 1.782 | 534 |
| *HIF2A* rs6756667 (AG+GG vs AA) | .977 | 1.005 | .725 | 1.392 | 534 |
| *HIF2A* rs1868086 (GT+TT vs GG) | .821 | 1.036 | .764 | 1.403 | 533 |
| *HIF2A* rs6712143 (AG+GG vs AA) | .989 | 1.002 | .748 | 1.342 | 534 |
| *HIF2A* rs10176396 (CT+TT vs CC) | .551 | 1.093 | .816 | 1.463 | 534 |
| *HIF2A* rs1374748 (GT+TT vs GG) | .145 | 1.277 | .919 | 1.773 | 534 |
| *HIF2A* rs3768728 (TC+CC vs TT) | .831 | .964 | .688 | 1.350 | 534 |
| *HIF2A* rs2346176 (TC+CC vs TT) | .775 | .957 | .711 | 1.290 | 534 |
| *HIF2A* rs10178633 (GA+AA vs GG) | .952 | .990 | .726 | 1.351 | 534 |
| *HIF2A* rs7594912 (AC+CC vs AA) | .467 | 1.126 | .818 | 1.548 | 534 |
| *HIF2A* rs7557402 (GC+CC vs GG) | .905 | .981 | .716 | 1.344 | 534 |
| *HIF2A* rs7571218 (GA+AA vs GG) | .515 | 1.105 | .818 | 1.493 | 534 |
| *LOX* rs3792802 (GA+AA vs GG) | .224 | 1.208 | .891 | 1.639 | 533 |
| *LOX* rs1800449 (GA+AA vs GG) | .217 | 1.210 | .894 | 1.637 | 534 |
| *CXCL12* rs2839695 (TC+CC vs TT) | .147 | .794 | .581 | 1.085 | 534 |
| *HIF1A* rs2301106 (TC+CC vs TT) | .951 | .989 | .706 | 1.386 | 534 |
| *HIF1A* rs12434438 (AG+GG vs AA) | .302 | 1.167 | .870 | 1.566 | 534 |
| *HIF1A* rs2057482 (CT+TT vs CC) | .815 | .962 | .693 | 1.335 | 534 |
| *HIF2B* rs12591286 (AG+AA vs GG) | .283 | .852 | .635 | 1.142 | 534 |
| *HIF2B* rs8041826 (AG+GG vs AA) | .866 | 1.027 | .751 | 1.406 | 534 |
| *HIF2B* rs1446337 (AG+AA vs GG) | .067 | .756 | .560 | 1.020 | 534 |
| *HIF2B* rs12593988 (GA+AA vs GG) | **.024** | .690 | .500 | .952 | 534 |
| *HIF2B* rs3848206 (AG+AA vs GG) | .700 | 1.070 | .760 | 1.506 | 533 |
| *HIF2B* rs3848207 (AG+AA vs GG) | .315 | 1.183 | .852 | 1.642 | 534 |
| *HIF2B* rs7172914 (CT+TT vs CC) | .141 | 1.250 | .929 | 1.681 | 534 |
| *HIF2B* rs10431813 (GA+AA vs GG) | .296 | .854 | .636 | 1.148 | 532 |
| *HIF2B* rs3910982 (TG+TT vs GG) | .149 | .807 | .603 | 1.080 | 534 |
| *HIF2B* rs11635014 (CT+TT vs CC) | .794 | 1.042 | .767 | 1.414 | 534 |
| *HIF2B* rs1020398 (TC+CC vs TT) | .467 | .897 | .670 | 1.202 | 534 |
| *HIF2B* rs11633642 (GA+AA vs GG) | .283 | .853 | .637 | 1.141 | 534 |
| *HIF2B* rs7184010 (CT+TT vs CC) | .411 | 1.130 | .844 | 1.514 | 533 |
| *HIF2B* rs3848170 (CT+TT vs CC) | .660 | 1.084 | .758 | 1.550 | 534 |
| *HIF2B* rs4778791 (GA+AA vs GG) | .211 | .816 | .594 | 1.122 | 534 |
| *HIF2B* rs8034535 (AG+GG vs AA) | .528 | 1.108 | .805 | 1.526 | 534 |
| *HIF2B* rs895442 (CT+TT vs CC) | .990 | 1.002 | .696 | 1.444 | 534 |
| *HIF2B* rs1037124 (GA+AA vs GG) | .462 | 1.125 | .822 | 1.539 | 534 |
| *HIF2B* rs1374213 (TC+CC vs TT) | .883 | 1.024 | .745 | 1.408 | 533 |
| *HIF2B* rs3901896 (CT+TT vs CC) | .303 | .854 | .633 | 1.153 | 534 |
| *HIF2B* rs2278709 (CT+TT vs CC) | .322 | .863 | .645 | 1.155 | 534 |
| *HIF2B* rs8028295 (CT+TT vs CC) | .623 | .925 | .677 | 1.263 | 534 |
| *HIF2B* rs4609803 (GA+AA vs GG) | .136 | 1.248 | .933 | 1.668 | 534 |
| *HIF2B* rs4778800 (TG+TT vs GG) | .882 | 1.023 | .761 | 1.375 | 534 |
| *HIF2B* rs7178902 (TC+CC vs TT) | .889 | 1.022 | .749 | 1.395 | 534 |
| *HIF2B* rs4238521 (GA+AA vs GG) | .460 | 1.124 | .824 | 1.534 | 534 |
| *HIF2B* rs4331301 (GA+AA vs GG) | .907 | 1.018 | .754 | 1.374 | 534 |
| *HIF2B* rs4778600 (TG+TT vs GG) | .992 | 1.002 | .720 | 1.393 | 533 |
| *HIF2B* rs11856676 (CT+TT vs CC) | .666 | .933 | .681 | 1.278 | 534 |
| *HIF2B* rs4238522 (TC+CC vs TT) | .880 | 1.024 | .754 | 1.390 | 534 |
| *HIF2B* rs4074666 (CT+TT vs CC) | .316 | .848 | .614 | 1.171 | 534 |
| *HIF2B* rs11635554 (AG+GG vs AA) | .844 | 1.030 | .769 | 1.378 | 534 |
| *HIF2B* rs4778818 (AG+GG vs AA) | .922 | 1.015 | .758 | 1.359 | 534 |
| *HIF2B* rs4778819 (CT+TT vs CC) | .843 | .971 | .726 | 1.299 | 534 |
| *HIF2B* rs7403706 (TC+CC vs TT) | .535 | 1.100 | .814 | 1.487 | 534 |
| *HIF2B* rs6495509 (GA+AA vs GG) | .578 | 1.088 | .808 | 1.466 | 534 |
| *HIF2B* rs8039725 (AG+GG vs AA) | .762 | .954 | .702 | 1.296 | 534 |
| *HIF2B* rs8033706 (TC+CC vs TT) | .089 | .753 | .542 | 1.044 | 478 |
| *HIF2B* rs4301984 (GA+AA vs GG) | .937 | 1.012 | .751 | 1.365 | 534 |
| *HIF2B* rs4459508 (GA+AA vs GG) | .876 | .977 | .730 | 1.307 | 534 |
| *HIF3A* rs2072491 (CT+TT vs CC) | .119 | .735 | .499 | 1.082 | 534 |
| *HIF3A* rs757638 (GA+AA vs GG) | .470 | .885 | .634 | 1.234 | 534 |
| *HIF3A* rs12461322 (GA+AA vs GG) | .345 | 1.184 | .834 | 1.682 | 534 |
| *HIF3A* rs887946 (AG+GG vs AA) | .786 | 1.041 | .778 | 1.394 | 534 |
| *HIF3A* rs3764610 (CT+TT vs CC) | .164 | 1.244 | .915 | 1.693 | 534 |
| *HIF3A* rs11665853 (AG+GG vs AA) | .830 | 1.040 | .725 | 1.494 | 534 |
| Sex (male vs female) | **.012** | 1.494 | 1.093 | 2.044 | 534 |
| Histology (mucinous vs non-mucinous) | .884 | .966 | .607 | 1.538 | 534 |
| Location (rectum vs colon) | .192 | 1.220 | .905 | 1.646 | 534 |
| Stage | **<.001** |  |  |  | 534 |
| Stage (II vs I) | .183 | 1.446 | .840 | 2.491 |  |
| Stage (III vs I) | **.004** | 2.201 | 1.290 | 3.754 |  |
| Stage (IV vs I) | **<.001** | 10.074 | 5.731 | 17.708 |  |
| Vascular invasion (+ vs -) | **<.001** | 1.714 | 1.266 | 2.319 | 495 |
| Lymphatic invasion (+ vs -) | **.003** | 1.571 | 1.164 | 2.122 | 492 |
| Familial risk (high/moderate vs low) | .640 | 1.072 | .801 | 1.436 | 534 |
| MSI status ( MSI-H vs MSS/MSI-l) | **<.001** | .232 | .103 | .525 | 512 |
| BRAF mutation status (+ vs -) | .395 | .794 | .466 | 1.351 | 483 |
| Age | .405 | 1.007 | .991 | 1.023 | 534 |
| Grade (poorly/ undifferentiated vs well/moderately differentiated) | .664 | .874 | .475 | 1.608 | 530 |

**Table S9.** Univariate analysis results for DFS (phase II)

| **Variables** | **p-value** | **HR** | **95% CI for HR** | | **n** |
| --- | --- | --- | --- | --- | --- |
|  |  |  | **Lower** | **Upper** |  |
| *HIF1B* rs10847 (AG+AA vs GG) | .359 | .881 | .672 | 1.155 | 533 |
| *HIF1B* rs2228099 (GC+CC vs GG) | .972 | .995 | .758 | 1.306 | 533 |
| *HIF1B* rs11204737 (TC+CC vs TT) | .819 | 1.034 | .778 | 1.373 | 533 |
| *HIF2A* rs1867785 (AG+AA vs GG) | .749 | 1.046 | .794 | 1.379 | 533 |
| *HIF2A* rs2121266 (AC+CC vs AA) | .740 | .954 | .723 | 1.259 | 532 |
| *HIF2A* rs17034950 (AG+AA vs GG) | .767 | 1.042 | .795 | 1.364 | 533 |
| *HIF2A* rs9973653 (TG+TT vs GG) | .736 | 1.047 | .800 | 1.371 | 533 |
| *HIF2A* rs4953342 (AG+GG vs AA) | .294 | 1.155 | .882 | 1.513 | 533 |
| *HIF2A* rs1868089 (TC+CC vs TT) | .639 | .930 | .685 | 1.262 | 533 |
| *HIF2A* rs6758592 (TC+CC vs TT) | .370 | 1.145 | .851 | 1.541 | 533 |
| *HIF2A* rs12614710 (TG+TT vs GG) | .654 | 1.072 | .791 | 1.453 | 533 |
| *HIF2A* rs4953352 (TC+CC vs TT) | **.009** | 1.574 | 1.122 | 2.207 | 533 |
| *HIF2A* rs4953353 (TG+TT vs GG) | .387 | 1.129 | .858 | 1.485 | 533 |
| *HIF2A* rs2346175 (TC+CC vs TT) | .338 | 1.159 | .858 | 1.565 | 533 |
| *HIF2A* rs6756667 (AG+GG vs AA) | .645 | .932 | .692 | 1.256 | 533 |
| *HIF2A* rs1868086 (GT+TT vs GG) | .713 | .948 | .714 | 1.259 | 532 |
| *HIF2A* rs6712143 (AG+GG vs AA) | .806 | .967 | .737 | 1.267 | 533 |
| *HIF2A* rs10176396 (CT+TT vs CC) | .873 | .978 | .747 | 1.281 | 533 |
| *HIF2A* rs1374748 (GT+TT vs GG) | .395 | 1.144 | .839 | 1.558 | 533 |
| *HIF2A* rs3768728 (TC+CC vs TT) | .687 | .937 | .684 | 1.284 | 533 |
| *HIF2A* rs2346176 (TC+CC vs TT) | .728 | .952 | .724 | 1.254 | 533 |
| *HIF2A* rs10178633 (GA+AA vs GG) | .736 | .952 | .716 | 1.266 | 533 |
| *HIF2A* rs7594912 (AC+CC vs AA) | .973 | 1.005 | .752 | 1.343 | 533 |
| *HIF2A* rs7557402 (GC+CC vs GG) | .431 | .891 | .668 | 1.188 | 533 |
| *HIF2A* rs7571218 (GA+AA vs GG) | .931 | .988 | .750 | 1.301 | 533 |
| *LOX* rs3792802 (GA+AA vs GG) | .173 | 1.216 | .917 | 1.613 | 532 |
| *LOX* rs1800449 (GA+AA vs GG) | .251 | 1.179 | .890 | 1.561 | 533 |
| *CXCL12* rs2839695 (TC+CC vs TT) | .288 | .857 | .644 | 1.140 | 533 |
| *HIF1A* rs2301106 (TC+CC vs TT) | .643 | 1.076 | .790 | 1.465 | 533 |
| *HIF1A* rs12434438 (AG+GG vs AA) | .070 | 1.285 | .980 | 1.686 | 533 |
| *HIF1A* rs2057482 (CT+TT vs CC) | .724 | 1.055 | .783 | 1.423 | 533 |
| *HIF2B* rs12591286 (AG+AA vs GG) | .354 | .879 | .670 | 1.154 | 533 |
| *HIF2B* rs8041826 (AG+GG vs AA) | .370 | 1.140 | .855 | 1.520 | 533 |
| *HIF2B* rs1446337 (AG+AA vs GG) | .117 | .802 | .609 | 1.057 | 533 |
| *HIF2B* rs12593988 (GA+AA vs GG) | **.042** | .736 | .548 | .988 | 533 |
| *HIF2B* rs3848206 (AG+AA vs GG) | .639 | 1.079 | .786 | 1.482 | 532 |
| *HIF2B* rs3848207 (AG+AA vs GG) | .253 | 1.193 | .882 | 1.614 | 533 |
| *HIF2B* rs7172914 (CT+TT vs CC) | .134 | 1.234 | .937 | 1.625 | 533 |
| *HIF2B* rs10431813 (GA+AA vs GG) | .536 | .917 | .698 | 1.205 | 531 |
| *HIF2B* rs3910982 (TG+TT vs GG) | .258 | .856 | .654 | 1.121 | 533 |
| *HIF2B* rs11635014 (CT+TT vs CC) | .234 | 1.191 | .893 | 1.587 | 533 |
| *HIF2B* rs1020398 (TC+CC vs TT) | .999 | 1.000 | .763 | 1.310 | 533 |
| *HIF2B* rs11633642 (GA+AA vs GG) | .134 | .814 | .621 | 1.065 | 533 |
| *HIF2B* rs7184010 (CT+TT vs CC) | .643 | .938 | .715 | 1.231 | 532 |
| *HIF2B* rs3848170 (CT+TT vs CC) | .970 | 1.006 | .720 | 1.407 | 533 |
| *HIF2B* rs4778791 (GA+AA vs GG) | .253 | .841 | .626 | 1.131 | 533 |
| *HIF2B* rs8034535 (AG+GG vs AA) | .352 | 1.152 | .855 | 1.551 | 533 |
| *HIF2B* rs895442 (CT+TT vs CC) | .774 | 1.050 | .751 | 1.468 | 533 |
| *HIF2B* rs1037124 (GA+AA vs GG) | .833 | .969 | .719 | 1.304 | 533 |
| *HIF2B* rs1374213 (TC+CC vs TT) | .645 | 1.072 | .797 | 1.442 | 532 |
| *HIF2B* rs3901896 (CT+TT vs CC) | .447 | .897 | .678 | 1.186 | 533 |
| *HIF2B* rs2278709 (CT+TT vs CC) | .797 | .965 | .737 | 1.264 | 533 |
| *HIF2B* rs8028295 (CT+TT vs CC) | .593 | .924 | .692 | 1.234 | 533 |
| *HIF2B* rs4609803 (GA+AA vs GG) | .283 | 1.159 | .885 | 1.518 | 533 |
| *HIF2B* rs4778800 (TG+TT vs GG) | .581 | .925 | .702 | 1.219 | 533 |
| *HIF2B* rs7178902 (TC+CC vs TT) | .529 | 1.097 | .821 | 1.466 | 533 |
| *HIF2B* rs4238521 (GA+AA vs GG) | .473 | 1.111 | .833 | 1.483 | 533 |
| *HIF2B* rs4331301 (GA+AA vs GG) | .428 | 1.119 | .847 | 1.480 | 533 |
| *HIF2B* rs4778600 (TG+TT vs GG) | .418 | .879 | .644 | 1.200 | 532 |
| *HIF2B* rs11856676 (CT+TT vs CC) | .473 | 1.115 | .828 | 1.501 | 533 |
| *HIF2B* rs4238522 (TC+CC vs TT) | .108 | 1.267 | .950 | 1.691 | 533 |
| *HIF2B* rs4074666 (CT+TT vs CC) | .822 | .966 | .714 | 1.307 | 533 |
| *HIF2B* rs11635554 (AG+GG vs AA) | .862 | .976 | .745 | 1.280 | 533 |
| *HIF2B* rs4778818 (AG+GG vs AA) | .795 | .965 | .736 | 1.265 | 533 |
| *HIF2B* rs4778819 (CT+TT vs CC) | .998 | 1.000 | .764 | 1.310 | 533 |
| *HIF2B* rs7403706 (TC+CC vs TT) | .890 | .980 | .739 | 1.300 | 533 |
| *HIF2B* rs6495509 (GA+AA vs GG) | .719 | .950 | .719 | 1.255 | 533 |
| *HIF2B* rs8039725 (AG+GG vs AA) | .225 | .837 | .628 | 1.116 | 533 |
| *HIF2B* rs8033706 (TC+CC vs TT) | **.023** | .704 | .521 | .953 | 477 |
| *HIF2B* rs4301984 (GA+AA vs GG) | .469 | .902 | .683 | 1.192 | 533 |
| *HIF2B* rs4459508 (GA+AA vs GG) | .321 | .872 | .665 | 1.143 | 533 |
| *HIF3A* rs2072491 (CT+TT vs CC) | .089 | .732 | .512 | 1.048 | 533 |
| *HIF3A* rs757638 (GA+AA vs GG) | .396 | .875 | .644 | 1.190 | 533 |
| *HIF3A* rs12461322 (GA+AA vs GG) | .512 | 1.117 | .802 | 1.557 | 533 |
| *HIF3A* rs887946 (AG+GG vs AA) | .971 | 1.005 | .767 | 1.316 | 533 |
| *HIF3A* rs3764610 (CT+TT vs CC) | .230 | 1.193 | .894 | 1.592 | 533 |
| *HIF3A* rs11665853 (AG+GG vs AA) | .965 | .992 | .706 | 1.396 | 533 |
| Sex (male vs female) | **.009** | 1.472 | 1.102 | 1.967 | 533 |
| Histology (mucinous vs non-mucinous) | .768 | .937 | .608 | 1.445 | 533 |
| Location (rectum vs colon) | **.028** | 1.361 | 1.033 | 1.793 | 533 |
| Stage | **<.001** |  |  |  | 533 |
| Stage (II vs I) | .258 | 1.315 | .818 | 2.115 |  |
| Stage (III vs I) | **.002** | 2.120 | 1.331 | 3.375 |  |
| Stage (IV vs I) | **<.001** | 5.688 | 3.428 | 9.436 |  |
| Vascular invasion (+ vs -) | **.001** | 1.633 | 1.232 | 2.162 | 494 |
| Lymphatic invasion (+ vs -) | **.003** | 1.524 | 1.153 | 2.014 | 491 |
| Familial risk (high/moderate vs low) | .286 | 1.159 | .884 | 1.519 | 533 |
| MSI status ( MSI-H vs MSS/MSI-l) | **.001** | .345 | .183 | .652 | 511 |
| BRAF mutation status (+ vs -) | .645 | .895 | .557 | 1.437 | 483 |
| Age | .847 | 1.001 | .987 | 1.016 | 533 |
| Grade (poorly/ undifferentiated vs well/moderately differentiated) | .477 | .809 | .452 | 1.450 | 529 |
